# Inhibition of FAM46/TENT5 activity by BCCIPα adopting a unique fold

**DOI:** 10.1126/sciadv.adf5583

**Published:** 2023-04-05

**Authors:** Shun Liu, Hua Chen, Yan Yin, Defen Lu, Guoming Gao, Jie Li, Xiao-Chen Bai, Xuewu Zhang

**Affiliations:** ^1^ Department of Pharmacology, University of Texas Southwestern Medical Center, Dallas, TX, USA.; ^2^ Department of Biophysics, University of Texas Southwestern Medical Center, Dallas, TX, USA.; ^3^ Department of Cell Biology, University of Texas Southwestern Medical Center, Dallas, TX, USA.

## Abstract

The FAM46 (also known as TENT5) proteins are noncanonical poly(A) polymerases (PAPs) implicated in regulating RNA stability. The regulatory mechanisms of FAM46 are poorly understood. Here, we report that the nuclear protein BCCIPα, but not the alternatively spliced isoform BCCIPβ, binds FAM46 and inhibits their PAP activity. Unexpectedly, our structures of the FAM46A/BCCIPα and FAM46C/BCCIPα complexes show that, despite sharing most of the sequence and differing only at the C-terminal portion, BCCIPα adopts a unique structure completely different from BCCIPβ. The distinct C-terminal segment of BCCIPα supports the adoption of the unique fold but does not directly interact with FAM46. The β sheets in BCCIPα and FAM46 pack side by side to form an extended β sheet. A helix-loop-helix segment in BCCIPα inserts into the active site cleft of FAM46, thereby inhibiting the PAP activity. Our results together show that the unique fold of BCCIPα underlies its interaction with and functional regulation of FAM46.

## Teaser

BCCIPɑ, adopting a completely different structure from its close relative BCCIPβ, inhibits the activity of the FAM46 proteins.

## INTRODUCTION

Family with sequence similarity 46 (FAM46) contains four highly conserved members FAM46A, FAM46B, FAM46C, and FAM46D (*1*, *2*). They are also known as terminal nucleotidyltransferases 5 (TENT5A, TENT5B, TENT5C, and TENT5D), referring to their noncanonical poly(A) polymerase (PAP) activity (*1*, *3*, *4*). Malfunction of the FAM46 proteins is associated with a number of diseases. Notably, loss-of-function mutation of FAM46C occurs frequently in multiple myeloma, strongly suggesting a tumor-suppressive role for FAM46C (*3*, *5*–*7*). Mutations of FAM46A have been linked to non–small cell lung cancer, familial autosomal recessive retinitis pigmentosa, and bone abnormalities (*8*–*12*). The wide variety of diseases associated with FAM46 mutations suggests important and diverse biological functions of these proteins. FAM46B is expressed in human preimplantation embryos and pluripotent stem cells and is essential for survival of embryonic stem cells (*4*). FAM46C and FAM46D have been shown to play critical roles in sperm development (*13*, *14*).

Recent studies have been directed at understanding the molecular pathways and mechanisms underlying the functions of FAM46 proteins, especially the tumor suppressor function of FAM46C in multiple myeloma. It has been shown that the PAP activity of FAM46 enhances stability of mRNAs by lengthening their poly(A) tails, which appears important for the roles of FAM46 in tumor suppression and adaptive and innate immunity (*3*, *5*, *15*, *16*). mRNAs encoding endoplasmic reticulum (ER)–targeted proteins are enriched in the pool of mRNAs stabilized by FAM46C, which depends on the interaction with the ER membrane protein fibronectin type III domain-containing protein (FNDC3) (*15*). Through this mechanism, FAM46C is proposed to raise the ER secretory capacity to abnormally high levels, leading to reactive oxygen species (ROS) production, adenosine triphosphate (ATP) shortage, and ultimately cell death of multiple myeloma (*15*). In addition, FAM46C seems to play a role in stabilizing immunoglobulin mRNA by catalyzing its polyadenylation and thereby boosts antibody production by B cells (*17*, *18*). However, another study provided evidence that the FAM46C/FNDC3 complex regulates protein secretion independently of the PAP activity (*19*). This study also suggested that FAM46C down-regulates autophagy through its interaction with FNDC3 and the autophagic receptor p62, and thereby promotes apoptosis of multiple myeloma through accumulation of intracellular protein aggregation (*19*). In addition, two studies have shown that the FAM46C protein interacts directly with and may regulate the activity of polo-like kinase 4 (Plk4), the master regulator of centrosome duplication, which is essential for the formation of the mitotic spindle for proper chromosome segregation in cell division (*5*, *20*). The interaction between FAM46C and Plk4 suggests that the regulation of centrosome duplication and consequently cell cycle by FAM46C contributes to their tumor-suppressive roles (*5*, *20*).

One consensus from these previous studies is that the PAP activity of the FAM46 proteins is of great importance to the cellular functions of these proteins. Mutations of FAM46 associated with multiple myeloma or other diseases mostly cause loss of the PAP activity or down-regulation of the protein levels (*5*, *21*). Proper regulation of the PAP activity of the FAM46 proteins is therefore expected to be important for development and tissue homeostasis. However, very little is known about how the PAP activity of FAM46 is regulated in cells under physiological conditions in the absence of mutations. Plk4, despite binding at the edge of the FAM46C active site, does not alter the enzymatic activity of FAM46C (*5*). To date, there is no evidence that other binding partners of FAM46 directly regulate their PAP activity.

To explore the regulatory mechanisms of the FAM46 PAP activity, we used a proteomics approach to identify additional proteins that interact with human FAM46C. These experiments led to the discovery that the FAM46 family members interact specifically with human BRCA2 and CDKN1A interaction protein α (BCCIPα; also known as p21 and CDK-associated protein 1α, TOK1α), but not the closely related BCCIPβ (*22*, *23*). Human BCCIPα and BCCIPβ share the identical 258-residue N-terminal portion but differ at their C-terminal portions, as a result of alternative splicing of the same transcript (*22*–*24*). The C-terminal 64 residues (residues 259 to 322) in BCCIPα are encoded by exons 8 and 9 of the BCCIP gene, whereas the 56-residue C-terminal region of BCCIPβ is encoded by exon 7 (*24*). BCCIPβ has been shown to be involved in many processes, including DNA repair, chromosome stability, cell cycle regulation, and ribosome biogenesis (*25*–*31*). BCCIPα, however, does not have most of these functions, except that it appears to share with BCCIPβ the ability of binding BRCA2 and CDKN1A (*22*, *23*, *31*). While BCCIPβ is present in lower organisms such as yeast (named BCP1) (*32*), BCCIPα has only been identified in primates and a few other species (*23*). Therefore, the cellular functions of BCCIPα remain largely unknown.

We find that the binding of BCCIPα inhibits the PAP activity of FAM46, providing a mechanism for regulating FAM46 in cells and establishing a previously unknown molecular pathway that underlies the biological function of BCCIPα. We report the crystal structures of the FAM46A/BCCIPα complex, as well as the cryo–electron microscopy (cryo-EM) structure of the FAM46C/BCCIPα complex. Strikingly, the structures show that BCCIPα adopts a structural fold that is completely different from BCCIPβ, which explains the selective interaction of FAM46 with BCCIPα, but not BCCIPβ. The structures also reveal how BCCIPα inhibits the enzymatic activity of FAM46.

## RESULTS

### Interaction of BCCIPα, but not BCCIPβ, with FAM46 in cells

**Fig. 1.**
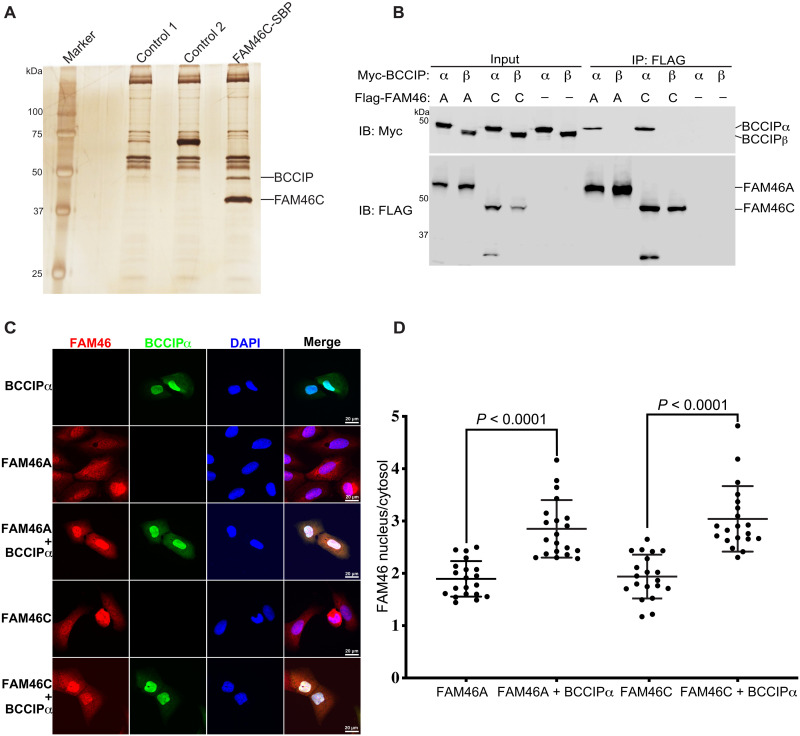
Specific interaction between FAM46 and BCCIPα in cells. (**A**) FAM46C, but not unrelated control proteins, expressed in HEK293 cells pulled down a protein of ~48 kDa, which was identified as BCCIP by mass spectrometry. (**B**) Coimmunoprecipitation results showing that both FAM46A and FAM46C interacted with BCCIPα, but not BCCIPβ, in HEK293 cells. (**C**) BCCIPα recruits both FAM46A and FAM46C into the nucleus in U2OS cells. (**D**) Quantification of the ratio of FAM46A and FAM46C between the nucleus and cytosol in cells from (C). Each dot represents the nucleus/cytosol amount ratio of FAM46 in one cell. The horizontal bars are the mean and SDs. *P* values are calculated using two-tailed Student’s *t* test. The results shown are representatives of three biological replicates.

To identify proteins interacting with FAM46C, we expressed human FAM46C with a FLAG and streptavidin-binding peptide (SBP) tandem tag in human embryonic kidney (HEK) 293 cells. Proteins coimmunoprecipitated with FLAG-SBP–tagged FAM46C were resolved on SDS–polyacrylamide gel electrophoresis (SDS-PAGE), which showed a prominent band at ~48 kDa that was absent in control samples (Fig. 1A). Mass spectrometric analyses of this band identified it as human BCCIPα or BCCIPβ. The mass spectrometry result identified four unique peptides (^9^AVESGVPQPPDPPVQR^24^, ^77^LLQQLFLK^84^, ^160^SMVEQLDK^167^, and ^225^TFVEAGKNNSK^235^) from BCCIP, which covered ~17% of the N-terminal common region of BCCIPα and BCCIPβ. However, no C-terminal peptides of BCCIP were recovered, and therefore, it was not clear whether FAM46C pulled down BCCIPα, BCCIPβ, or both. The theoretical molecular weight of BCCIPα and BCCIPβ is ~35 and 34 kDa, respectively, although they migrated slower than expected on SDS-PAGE, with an apparent molecular weight close to 50 kDa (*22*, *23*). A recent study using a similar approach also identified the FAM46C/BCCIP interaction, but assigned the interaction specifically to BCCIPβ (*3*).

To answer the question which isoform of BCCIP interacted with FAM46C, we coexpressed FAM46C with either BCCIPα or BCCIPβ in HEK293 cells and carried out coimmunoprecipitation experiments. As shown in Fig. 1B, FAM46C coimmunoprecipitated with BCCIPα, but not BCCIPβ, suggesting that the interaction with FAM46C is specific to BCCIPα. We noticed that FAM46C migrated as two bands when coexpressed with BCCIPα (Fig. 1B). The upper band was the full-length protein, while the lower band was likely a degradation product, suggesting that the binding of BCCIPα increased the structural flexibility of FAM46C and its vulnerability to protease cleavage (see more below). To establish the generality of the FAM46/BCCIPα interaction, we also carried out the coimmunoprecipitation experiments with FAM46A. The results confirmed that FAM46A specifically interacts with BCCIPα, but not BCCIPβ (Fig. 1B).

Previous studies have found that both BCCIPα and BCCIPβ have a nuclear localization signal (NLS) sequence, which drives their localization to the nucleus (*22*, *23*, *33*). Consistently, our immunofluorescence results showed that BCCIPα was mostly confined to the nucleus when ectopically expressed in U2OS cells (Fig. 1C). FAM46C, however, has a broad distribution in the cell but could be recruited to different compartments by its binding partners such as Plk4 and FNDC3 (*5*, *15*, *18*–*20*). Our results showed that both FAM46A and FAM46C expressed in U2OS cells were distributed in both the nucleus and cytoplasm, with a mean intensity ratio in the two compartments of 1.90 and 1.94, respectively (Fig. 1, C and D). However, they were strongly enriched in the nucleus when coexpressed with BCCIPα, with the mean nucleus/cytoplasm intensity ratio increased to 2.85 and 3.04 for FAM46A and FAM46C, respectively (Fig. 1, C and D). These results confirm the notion that BCCIPα and FAM46 can interact with each other in cells. In addition, these results suggest that BCCIPα may regulate the functions of the FAM46 proteins by sequestering them in the nucleus, although this potential mechanism needs to be further tested with endogenously expressed BCCIPα and FAM46.

### Direct interaction and inhibition of the PAP activity of FAM46 by BCCIPα

**Fig. 2.**
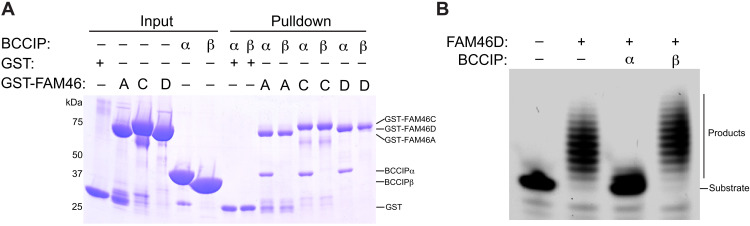
BCCIPα directly interacts with FAM46 and inhibits their PAP activity. (**A**) Purified GST-tagged FAM46A, FAM46C, and FAM46D interact with BCCIPα, but not BCCIPβ. (**B**) The PAP activity of FAM46D is inhibited by BCCIPα, but not BCCIPβ. The results shown are representatives of three biological replicates.

To test whether the interaction between FAM46 and BCCIP is direct, we carried out binding assays with purified proteins. The results showed that BCCIPα, but not BCCIPβ, was pulled down by FAM46C (Fig. 2A). In addition, FAM46A and FAM46D showed the same specific interaction with BCCIPα (Fig. 2A). These results together demonstrated that the direct interaction with BCCIPα is a conserved property of the FAM46 family members.

To investigate the function of the FAM46/BCCIPα interaction, we tested whether the interaction alters the PAP activity of FAM46 by using a gel shift assay that reports the elongation of the poly(A) tail of a fluorescence-labeled RNA substrate (*5*). We used FAM46D in this assay because the PAP activity of FAM46D is the highest among all the FAM46 family members and therefore more readily detectable (*3*, *5*). As shown in Fig. 2B, FAM46D catalyzed robust elongation of the poly(A) tail of the RNA substrate. The elongation was strongly inhibited in the presence of BCCIPα at a 1:1 molar ratio, whereas BCCIPβ at the same concentration showed no inhibitory effect (Fig. 2B). These results demonstrate that BCCIPα, but not BCCIPβ, can inhibit the PAP activity of FAM46 through the direct interaction.

### Crystallization and structure determination of BCCIPα in complex with FAM46A

**Fig. 3.**
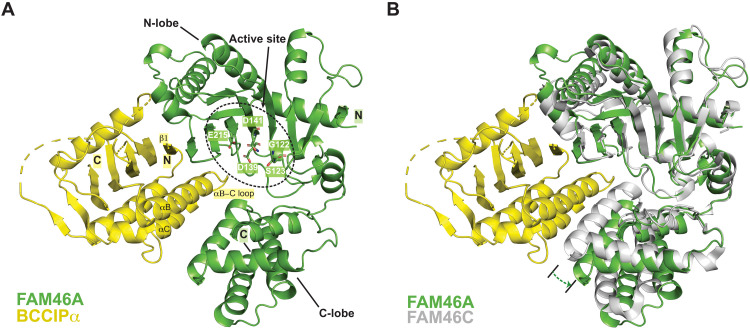
Crystal structure of the FAM46A/BCCIPα complex. (**A**) Overall structure of the FAM46A/BCCIPα-ΔL complex. Residues in the active site of FAM46A are highlighted. The structural elements in BCCIPα involved in binding to FAM46A are labeled. N and C denote the N and C termini of the proteins, respectively. (**B**) Structural comparison between FAM46A in the FAM46A/BCCIPα complex and FAM46C in the apo-state (PDB ID: 6W36). The structures are superimposed on the basis of the N-lobe of FAM46A and FAM46C. The dashed arrow indicates that the C-lobe of FAM46A in the complex rotates away from the N-lobe compared with that in apo-FAM46C.

To understand how BCCIPα binds the FAM46 proteins and inhibits their PAP activity, we sought to solve the crystal structure of a FAM46/BCCIPα complex. After extensive screening of various constructs of BCCIPα and the FAM46 family members, we were able to obtain crystals of the complex between FAM46A and BCCIPα-ΔS, which contains deletions in flexible loop regions (see Materials and Methods for details). We confirmed that BCCIPα-ΔS and BCCIPα-WT showed similar binding to FAM46A and inhibition of the PAP activity of FAM46D (fig. S1). We solved the structure of this complex to 3.5-Å resolution, revealing the binding mode between BCCIPα and FAM46A (fig. S2A). Strikingly, the structure showed that BCCIPα adopts a structural fold that is completely different from the structure of BCCIPβ published previously (see details below) (*34*, *35*). Analyses of this structure allowed us to design a new construct, BCCIPα-ΔL, containing a larger deletion (residues 231 to 280) of a long disordered loop (fig. S3), which did not noticeably affect the binding with FAM46A or the inhibition of the PAP activity (fig. S1). The positively charged NLS peptide (residues 235 to 243) of BCCIP is located in this long loop (fig. S3) (*22*, *33*). Our observation that BCCIPα can promote the nuclear localization of FAM46 is consistent with the structure of the complex where the NLS is available for driving their nuclear translocation as it is not occupied by the binding interface with FAM46. The FAM46A/BCCIPα-ΔL complex yielded better crystals. The structure of the FAM46A/BCCIPα-ΔL complex is very similar to the FAM46A/BCCIPα-ΔS complex, with the root mean square deviation (RMSD) of aligned Cα atoms of 1.3 Å, but reached higher resolution (3.2 Å) (Fig. 3A and fig. S2B). The following description of the FAM46A/BCCIPα structure will refer to this higher-resolution structure.

### Unique fold of BCCIPα

The structure of the FAM46A/BCCIPα complex for the first time revealed the fold of BCCIPα. Contrary to the expectation based on their sequence similarity (fig. S3), BCCIPα does not share the same structural fold as BCCIPβ. Notably, models of BCCIPα generated by AlphaFold without using structural templates and ESMFold that uses language model–based prediction closely resemble the structure of BCCIPβ, rather than that of BCCIPα solved here (fig. S4) (*34*–*38*). Furthermore, A DALI search of the protein structural database suggested that the fold of BCCIPα is unique, as the search failed to find any protein with substantial similarity to BCCIPα. The top hit in the DALI result is human serine palmitoyl transferase 1 [Protein Data Bank (PDB) ID: 7K0M], which has an RMSD of 9.9 Å for 62 aligned residues with BCCIPα (*39*).

**Fig. 4.**
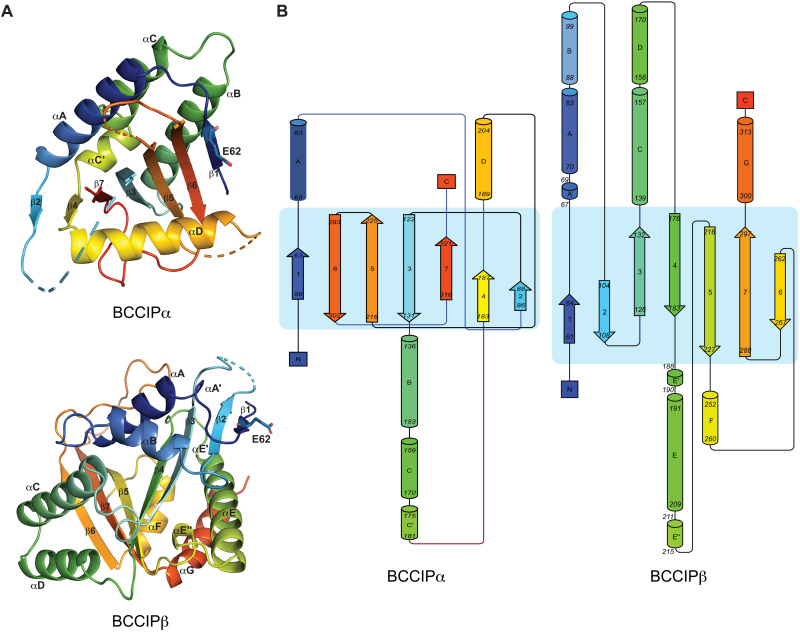
BCCIPα and BCCIPβ adopt different structural folds. (**A**) Comparison of the structure of human BCCIPα and BCCIPβ (PDB ID: 7KYS). The structures are colored with the rainbow color scheme from the N to C termini. The E62 residues in both structures are highlighted to illustrate the fact that strands β1 in BCCIPα and BCCIPβ use their opposite side to pack with their respective neighboring strands. (**B**) Topological diagrams of BCCIPα and BCCIPβ.

The previously published structures of BCCIPβ from human and yeast both exhibit a fold similar to GCN-5–related acetyltransferases, characterized by a splayed seven-stranded β sheet that is surrounded by several α helices on each side (Fig. 4A) (*34*, *35*). BCCIPβ is, however, unlikely an acetyltransferase because it lacks the catalytic residues conserved in those enzymes (*34*, *35*). BCCIPα also contains a central β sheet, but the order and directions of the β strands are entirely different from those in BCCIPβ (Fig. 4, A and B). The β strands in BCCIPβ are arranged largely in a sequential manner in space, with the order of strands presented as 1-2-3-4-5-7-6. The strands are mostly antiparallel, with the exception that strands β4 and β5 are parallel. In contrast, while the β sheet in BCCIPα also starts with strand β1 at one edge, the strands are arranged in space in the order of 1-6-5-3-7-4-2. Strands 1-6-5-3-7 are all antiparallel, whereas strands 7-4-2 are parallel. The C-terminal region (residues 296 to 322) in BCCIPα is predicted to be helical by secondary structure prediction. However, in our structure, this region forms two β strands, strands 6 (residues 296 to 303) and 7 (residues 316 to 320). These two strands constitute the core part of the β sheet and therefore play a critical role in adopting the unique fold of BCCIPα. The C-terminal segment in BCCIPβ instead forms strands β6 and β7 and helices F and G.

More detailed analyses showed that while strands β1 in both BCCIPα and BCCIPβ are placed at the edge of the β sheets, they use the opposite side to form hydrogen bonds with their respective neighboring β strands. This difference is evident in the structural comparison shown in Fig. 4A. Glu^62^ in strand β1 of BCCIPα uses its backbone amide group to form a hydrogen bond with the neighboring strand β6 in the central β sheet while leaving its carbonyl group exposed to solvent, which constitutes a part of the binding interface for FAM46 (see details below). In contrast, the carbonyl group in Glu^62^ of BCCIPβ is involved in hydrogen bond formation in the β sheet, whereas the amide group is at the outer edge and exposed to solvent.

The placements of the helices surrounding the β sheets are also different between BCCIPα and BCCIPβ. The seven helices in BCCIPβ surround the central part of the β sheets in a largely evenly distributed manner but leave the edge strands, including β1 and β6, partially or fully exposed to the solvent. In BCCIPα, one side of the β sheet is covered by three helices (A, B, and C). Helix D is the single helix that packs on top of the opposite side of the β sheet, with helix D oriented roughly perpendicular to the β strands. A notable feature of BCCIPα is that strand β1 is sandwiched between helices D and B, resulting in a three-layered structure at the edge that forms the binding interface for FAM46.

We tried but failed to obtain a structure of BCCIPα in the apo-state, leaving open the possibility that BCCIPα in the apo-state adopts the BCCIPβ-like fold but converts to the unique fold only upon binding to FAM46, as protein/protein interactions are known to be a trigger of fold switch for some fold-switching proteins (*40*). To address this question, we designed several mutations in both BCCIPα and BCCIPβ and examined their effects on the protein stability, by comparing the ratio of the proteins in the soluble versus insoluble fractions when they are expressed in *Escherichia coli*. On the basis of the structures, L96 and I106 are critical for the BCCIPβ fold, but they are in a disordered region in BCCIPα (fig. S5). Consistently, the L96E and I106E mutations abolished the soluble fraction of BCCIPβ while having no effect on the soluble expression of BCCIPα (fig. S5). In contrast, Y217 is a part of the hydrophobic core of BCCIPα but surface-exposed in BCCIPβ. The Y217E mutation reduced the soluble fraction of BCCIPα, whereas BCCIPβ was unaffected by this mutation. Last, we made a mutation of T316 at the unique C-terminal tail of BCCIPα, buried inside the β sheet in the crystal structure. The T316E mutation abolished soluble expression of BCCIPα. These results together strongly suggest that BCCIPα adopts its unique fold on its own and is not capable of adopting the BCCIPβ fold.

### Inhibition of the FAM46 PAP activity by BCCIPα through blocking the active site

Consistent with the published structures of FAM46B and FAM46C (*4*, *5*, *41*), FAM46A in the FAM46A/BCCIPα complex adopts the same bi-lobal structure, with the PAP enzymatic active site residues in the cleft between the two lobes (Fig. 3A). The N-terminal lobe contains a large β sheet that is covered by several α helices on one side, whereas the other side of the β sheet faces the catalytic site cleft, presenting the conserved catalytic residues, including Gly^122^, Ser^123^, Asp^139^, Asp^141^ (for binding ATP and Mg^2+^), and Glu^215^ (the catalytic base), to form the active site of the enzyme.

**Fig. 5.**
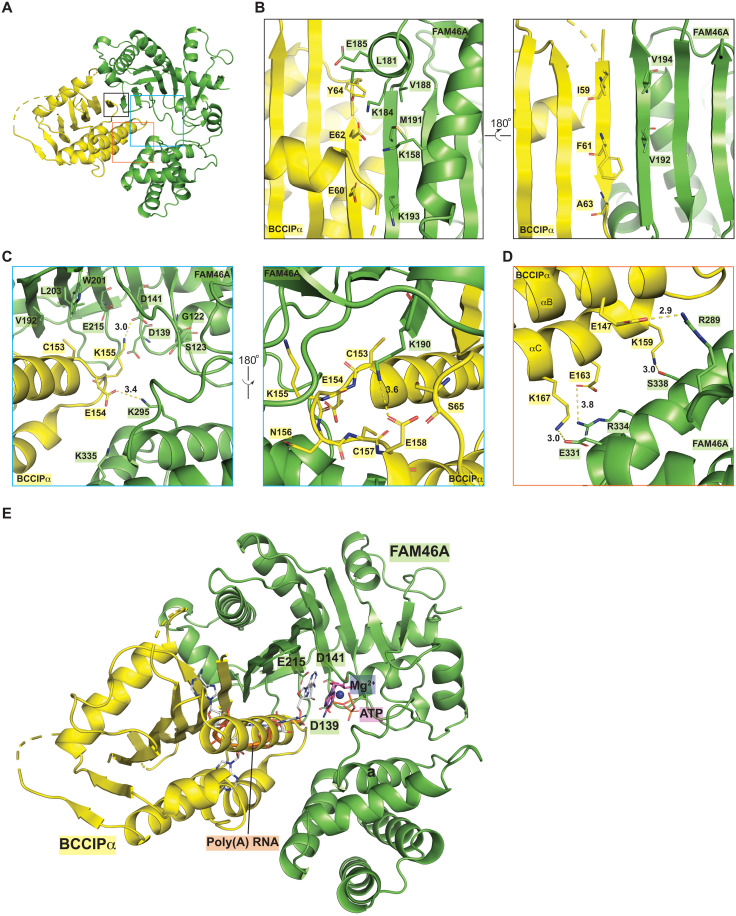
Detailed views of the binding interface between FAM46A and BCCIPα. (**A**) Overview of the structure of the FAM46A/BCCIPα complex. (**B** to **D**) Detailed views of the different parts of the binding interface. Each panel is an expanded view of the regions in the colored boxes in (A). (**E**) BCCIPα inhibits the PAP activity of FAM46A by blocking the substrate binding site of FAM46A. The ATP and RNA substrates are modeled on the basis of a structural superimposition of FAM46A with the structure of a yeast PAP in complex with ATP and poly(A)-RNA (PDB ID: 2Q66). On the basis of this model, it is evident that BCCIPα severely clashes with the poly(A)-RNA substrate of FAM46.

BCCIPα forms a large binding interface with the active site cleft of FAM46A, burying ~2600-Å^2^ solvent-accessible area (Figs. 3A and 5A). The strand at the outer edge of the active site cleft of FAM46A (residues 189 to 196) packs against strand β1 in BCCIPα in an antiparallel fashion, integrating the β sheets in the two proteins into one extended β sheet. In this regard, this interaction between BCCIPα and FAM46A is similar to that between Cryptic Polo-Box domain 1 (CPB1) in Plk4 and FAM46C reported previously (*5*). However, CPB1 of Plk4 leaves the active site in FAM46C unoccupied, and its binding does not affect the PAP activity of FAM46 (*5*). In contrast, as shown in Fig. 3A, helices B and C and the loop between them at the bottom of the β sheet in BCCIPα are wedged into the active site cleft in FAM46A. To accommodate BCCIPα, the C-terminal lobe of FAM46 rotates away substantially from the N-terminal lobe and creates a larger opening, as shown by a comparison of the complex structure with FAM46C in the apo-state (Fig. 3B). A superimposition of the complex structure with the structure of a PAP bound to RNA, ATP, and Mg^2+^ (PDB ID: 2q66) suggests that BCCIPα completely occupies the binding site for the RNA substrate in FAM46A (Fig. 5E) (*42*). These analyses therefore led to the model showing that BCCIPα inhibits the PAP activity of FAM46 by direct obstruction of the active site.

### Details of the binding interface between BCCIPα and FAM46A

Strand β1 in BCCIPα and the edge β strand in FAM46A form seven backbone hydrogen bonds to join the β sheets in the two proteins together (Figs. 3A and 5B). The binding interface is augmented by interactions mediated by side chains of residues in these two β strands. For example, extensive hydrophobic packing interactions are formed by Ile^59^, Phe^61^, Ala^63^, and Tyr^64^ in BCCIPα and Leu^181^, Val^188^, Met^191^, Val^192^, and Val^194^ in FAM46A. In addition, the interface contains several charge-charge interactions. Glu^60^ and Glu^62^ in BCCIPα makes charge complementary interactions with Lys^158^, Lys^184^, and Lys^193^ in FAM46A (Fig. 5B). On the interface close to the active site, hydrophobic interactions are formed by Cys^153^ in BCCIPα and Val^192^, Trp^201^, and Leu^203^ in FAM46A. Glu^154^ in BCCIPα interacts with Lys^295^ in FAM46A via a hydrogen bond. Lys^155^ at the tip of the loop between helices B and C in BCCIPα sticks deep into the active site and makes a direct interaction with the catalytic residues Asp^141^ and Glu^215^. Conversely, Lys^190^ in FAM46A contacts the side chain of Glu^158^ as well as a few main-chain carbonyls in the αB-αC loop in BCCIPα (Fig. 5C). Furthermore, the bottom surface of helices αB and αC makes a few polar contacts with the C-terminal lobe of FAM46A (Fig. 5D). It is likely that the charge-charge interactions not only contribute to the binding energy but also determine the specificity of the binding interface. Most of the residues in FAM46A involved in the binding interface are conserved in other FAM46 family members, which underlies the interaction of BCCIPα with all the FAM46 proteins.

All the residues in BCCIPα involved in binding FAM46 belong to the N-terminal portion of the sequence that is identical to BCCIPβ. In particular, strand β1 in BCCIPα, which makes the most contributions to the binding interface, is present in BCCIPβ. However, as mentioned above, the side of β1 that mediates the interaction with FAM46 in BCCIPα is instead involved in the formation of the β sheet within BCCIPβ itself. The residues forming helices αB and αC in BCCIPα (137 to 169) are also present in BCCIPβ, but they form two helices (D and E) that are placed far away from strand β1, preventing them from forming a binding interface together. Therefore, while the distinct C-terminal segment in BCCIPα underlies its specific interaction with FAM46, it does so indirectly by dictating the distinct structural fold, rather than directly mediating the interaction.

### Cryo-EM structure of the FAM46C/BCCIPα complex

**Fig. 6.**
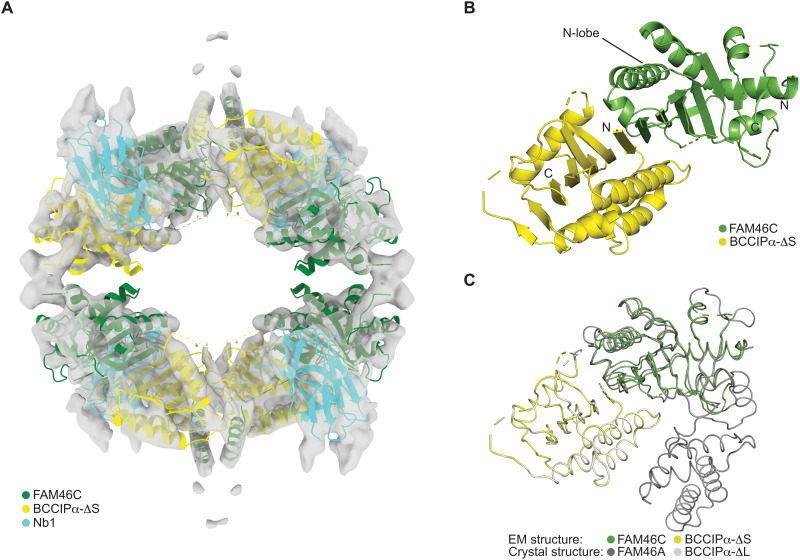
Cryo-EM structure of the FAM46C/BCCIPα/Nb.1 complex. (**A**) Cryo-EM map and the atomic model of the 4:4:4 complex. (**B**) Overview of one FAM46C/BCCIPα unit from the 4:4:4 complex. (**C**) Comparison of the FAM46C/BCCIPα cryo-EM structure with the FAM46A/BCCIPα crystal structure.

To confirm the unique structure of BCCIPα and establish the generality of its binding mode with the FAM46 proteins, we sought to solve the structure of BCCIPα in complex with another FAM46 family member. By screening a synthetic nanobody library (*43*), we identified a nanobody (referred to as Nb.1 thereafter) that could bind the FAM46C/BCCIPα-ΔS complex and induce its oligomerization as indicated by the profile of gel filtration chromatography (fig. S6). Cryo-EM images showed that a small portion of the FAM46C/BCCIPα-ΔS/Nb.1 complex formed filamentous oligomers resembling strings of Chinese lanterns. We solved the structure of this complex to a nominal resolution of 6.5 Å by treating each segment of the filaments as single particles (fig. S7). The cryo-EM map resolved secondary structural elements for model building through rigid-body docking and manual adjustments of the individual protein components (fig. S7). The final model of the complex has a 4:4:4 stoichiometry of the three proteins, formed by four units of the 1:1:1 FAM46C/BCCIPα/Nb.1 complex packing together with the D2 symmetry (Fig. 6). Nb.1 binds at the junction between BCCIPα and the N-terminal lobe of FAM46, but the details of the binding interface are not resolved by the cryo-EM map.

The atomic model of BCCIPα from the crystal structure could be unambiguously docked into the cryo-EM map, in which the central β sheet and the major α helices are clearly resolved (Fig. 6A and fig. S7F). The N-terminal lobe of FAM46C could also be docked into the map, which forms a binding interface with BCCIPα that is very similar to that in the crystal structure of the FAM46A/BCCIPα complex, as shown by the good superimposition of the FAM46 N-terminal lobes when BCCIPα molecules in the two complexes are aligned (Fig. 6C). These results provide strong validation for the unique structure of BCCIPα and the binding mode between BCCIPα and the FAM46 proteins as seen in the crystal structure.

The C-terminal lobe of FAM46C is not visible in the cryo-EM structure. As in the FAM46A/BCCIPα complex, the binding of BCCIPα to the active site cleft in FAM46C likely pushes the C-lobe away from the N-lobe, making the C-lobe more mobile. This increased structural flexibility may underlie the degradation of FAM46C when coexpressed with BCCIPα in cells (Fig. 1B). In addition, a comparison of the cryo-EM structure with the crystal structures of apo-FAM46C and the FAM46A/BCCIPα complex shows that Nb.1 partially occupies the position of the FAM46C C-terminal lobe (Fig. 6). This observation suggests that Nb.1 also contributes to the dislodging of the C-terminal lobe of FAM46C. There are two helices located near the two neighboring BCCIPα molecules in the complex. Additional density connecting to these two helices are present at the junction between the lantern-shaped units in the filaments of the FAM46C/BCCIPα-ΔS/Nb.1 complex (Fig. 6A). It is possible that this part of the structure is composed of structural elements from the C-terminal lobe of FAM46C, which is dislodged from the N-terminal lobe and changes its conformation to mediate the high-order oligomerization of the complex. Given that this conformation of FAM46C and the filamentous assembly are induced in part by an artificial nanobody, we do not assign them any physiological significance.

### Mutational analyses of the binding interface between BCCIPα and FAM46

**Fig. 7.**
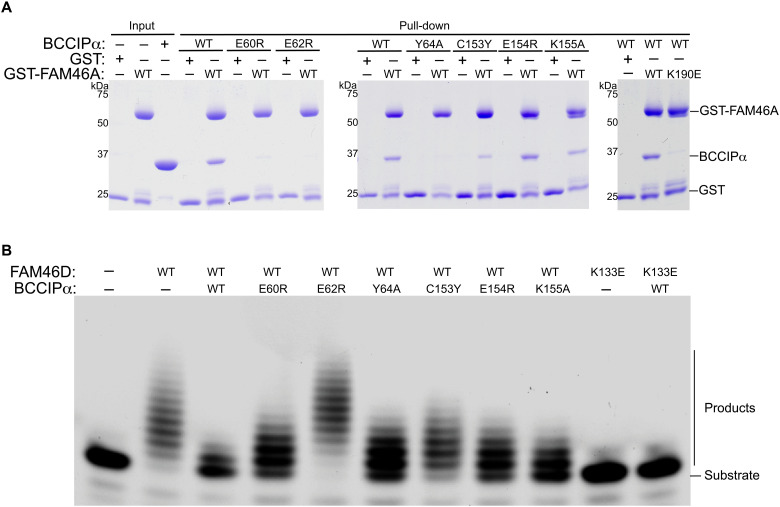
Mutational analyses of the FAM46/BCCIPα binding interface. (**A**) Effects of mutations in the binding interface on the binding between FAM46A and BCCIPα. (**B**) Effects of mutations in the binding interface on the inhibition of the FAM46D PAP activity by BCCIPα. The results shown are representatives of three biological replicates.

To confirm the binding mode between BCCIPα and FAM46 shown in the structures, we designed mutations to residues in the interface and tested their effects on the interaction using the pull-down assay. In this assay and the following PAP activity assay, we used BCCIPα without the loop deletions to ensure that the effects of the mutations are not confounded by the deletions. As shown in Fig. 7A, the E60R, E62R, and Y64A mutations in strand β1 of BCCIPα virtually abolished the binding to FAM46A. Similarly, the C153Y and K155A mutations in the loop between helices B and C of BCCIPα substantially reduced the binding to FAM46A. Conversely, the K190E mutation in the edge strand of FAM46A also abolished the FAM46A/BCCIPα interaction.

We also examined the effects of the interface mutations on the inhibition of the FAM46D PAP activity by BCCIPα. The results show that the E62R mutation in BCCIPα abolished its inhibition of the PAP activity (Fig. 7B). The E60R, Y64A, C153Y, E154R, and K155A mutations in BCCIPα reduced the inhibitory effect to various extent. We also tested the K133E mutation in FAM46D, corresponding to the K190E mutation in FAM46A, which abrogated the formation of the complex as shown in Fig. 7A. The K133E mutant of FAM46D did not show PAP activity in the absence or presence of BCCIPα. We reasoned that this residue may be required for binding of the RNA substrate as it is a positively charged residue located at the entrance of the active site of FAM46D. These results on the complex formation and the PAP activity together validate the binding mode between BCCIPα and FAM46 and the mechanism by which BCCIPα inhibits the FAM46 activity based on our structures.

## DISCUSSION

Little is currently known about the biological functions of BCCIPα since its identification as a binding partner of BRCA2 and the CDK2 regulator p21 over 2 decades ago (*22*, *23*). Both BCCIPα and BCCIPβ have been implicated in regulating DNA repair and cell cycle progression, which are linked to genome stability and cancer development, but their exact roles and mechanisms in these processes are not well understood (*22*, *23*, *30*, *33*, *44*). Previous studies have indicated that the functions of BCCIPα and BCCIPβ are different. BCCIPβ, but not BCCIPα, can bind the ribosomal protein RPL23 and act as its chaperone before its incorporation to the ribosome (*25*, *27*, *29*). In another study, Huhn *et al.* (*26*) found that BCCIPα was the dominant isoform localized at the centrosome and spindle pole, and BCCIPβ only showed weak association with the centrosome and the spindle pole. Our finding of the BCCIPα and FAM46 interaction further supports the notion that the functions of BCCIP are isoform specific. In addition to BCCIPα and BCCIPβ, the human genome encodes another BCCIP isoform as shown in the UniProt database, which have not been experimentally characterized. We attempted but failed at purifying this isoform of BCCIP and therefore could not test whether they interact with FAM46.

Understanding the isoform-specific functions of BCCIPα and BCCIPβ has been confounded by the facts that they share the identical N-terminal sequence and only differ at a relatively small C-terminal segment and that BCCIPα is not found in mice (*30*). Isoform-specific knockout in animal models is an ideal approach for dissecting the in vivo functions of protein isoforms from alternative splicing, which is hindered by the absence of BCCIPα in model organisms such as mice. Our study uncovers a novel function specific to BCCIPα and the underlying molecular basis by establishing that BCCIPα, but not BCCIPβ, interacts directly with the FAM46 proteins and inhibits their PAP activities. FAM46C has been shown to act as a tumor suppressor through several mechanisms, including by regulating RNA stability with its PAP activity and by interacting with proteins such as Plk4 (*3*, *5*, *15*, *19*, *20*). The interaction of BCCIPα with FAM46C and other FAM46 family members may be a part of a previously unknown signal pathway for regulating genome stability, cell cycle, and tumor development. Our findings set the stage for future studies to explore this pathway and understand the precise mechanisms underlying how BCCIPα and FAM46 work together in these processes.

Alternative splicing is a mechanism in eukaryotes for expanding the coding capacity of the genome and producing protein isoforms with different functions. Considering that BCCIPβ is conserved in diverse organisms from yeast to human, whereas BCCIPα has only been found in primates and a few other mammals, BCCIPα appears to have emerged later in evolution to carry out new functions in higher organisms. It has been suggested that the majority of the alternative splicing events generates protein isoforms that show relatively small structural differences (*45*, *46*). Insertion or deletion of a short segment in proteins as a result of alternative splicing often only causes local changes to the structure, while the global fold remains the same (*47*). To the best of our knowledge, our findings here represent the first case where the alternative splicing leads to two protein isoforms adopting completely different folds, which underlies their different specific protein interactions and functions. The different folds of BCCIPα and BCCIPβ are reminiscent of protein fold switching, where one protein can reversibly switch between two different native structures under different conditions (*40*). Protein fold switching is usually triggered by changes in pH or salt concentration, posttranslational modifications, or interactions with binding partners (*40*). In the case of BCCIP, the different sequences at the relatively short C-terminal region alter the free energy landscape to dictate the different folds of BCCIPα and BCCIPβ. The precise biophysical basis of this new type of fold switching requires further investigation.

## MATERIALS AND METHODS

### Antibodies and reagents

The following antibodies were used for immunoblotting or immunofluorescent analysis in this study: anti-FLAG (F1804, Sigma-Aldrich), anti-FLAG (rabbit, F7425, Sigma-Aldrich), anti-Myc (11667203001, Roche), anti-Myc (2276S, Cell Signaling Technology), anti-mouse immunoglobulin G (IgG) (H+L), F(ab′)_2_ fragment (Alexa Fluor 488 conjugate) (4408S, Cell Signaling Technology), goat anti-rabbit IgG (H+L) cross-adsorbed secondary antibody, cyanine3 (A10520, Invitrogen), and anti-rabbit IgG (H+L) (DyLight800 or DyLight680 conjugates) and anti-mouse IgG (H+L) (DyLight800 or DyLight680 conjugates, Cell Signaling Technology).

### Identification of BCCIP as a binding partner of FAM46 through cellular pulldown and mass spectrometry

A Flp-In HEK293 (R75007, Thermo Fisher Scientific) stable cell line expressing C-terminal FLAG-SBP–tagged FAM46C (residues 1 to 350) was generated with a modified pcDNA5/FRT vector (V601020, Thermo Fisher Scientific), according to the manufacturer’s instruction. Briefly, the Flp-in HEK293 cells were cotransfected with the pOG44 and FAM46C plasmids. Hygromycin was added into the medium to the final concentration of 100 μg/ml 48 hours after transfection to select the cells. FAM46C expression was confirmed with anti-FLAG antibody after hygromycin selection. The FAM46C protein was immunoprecipitated by streptavidin agarose beads (20359, Thermo Fisher Scientific), and the bounded proteins were eluted and separated by SDS-PAGE gel. The proteins were visualized by silver staining (LC6070, Thermo Fisher Scientific). The band of interest was cut, and proteins were identified with mass spectrometry analysis, performed by the Taplin Biological Mass Spectrometry Facility (Harvard University).

### Protein expression and purification

Gene fragments encoding the human FAM46 and BCCIP proteins were inserted into a modified pET28a vector that includes a cleavable N-terminal His_6_ tag for expression in *E. coli*. Fragments encoding the human FAM46 proteins were inserted into the pGEX-6P vector for production of glutathione *S*-transferase (GST)–tagged proteins. Point mutations were introduced with a Q5 site-directed mutagenesis kit (E0554, New England Biolabs) following the manufacturer’s protocols, and mutant constructs were confirmed by DNA sequencing.

Modified pET28a plasmids encoding target proteins were transformed into *E. coli* BL21 (DE3) competent cells. Cells were cultured in Terrific Broth medium at 37°C. Protein expression was induced by the addition of 0.2 mM isopropyl-β-d-thiogalactopyranoside (IPTG) at 16°C for overnight when OD_600_ (optical density at 600 nm) reached 3.0. For protein purification, cell pellets were resuspended in a buffer containing 50 mM tris-HCl (pH 7.5), 500 mM NaCl, and 10 mM imidazole and lysed with a cell homogenizer on ice. The lysate was centrifuged at 35,000*g* for 30 min. The supernatant was incubated with Ni^2+^–nitrilotriacetic acid agarose resin (30230, Qiagen) at 4°C for 2 hours with rotation. After incubation, resin was loaded onto a column and washed with a washing buffer [50 mM tris-HCl (pH 7.5), 500 mM NaCl, and 20 mM imidazole]. His_6_-tagged 3C protease was added for an overnight on-column cleavage at 4°C. After cleavage, target protein without tag was washed from the column with a washing buffer. Protein sample was diluted, loaded onto a Resource Q column equilibrated with a buffer containing 10 mM tris-HCl (pH 8.0), 10 mM NaCl, and 1 mM dithiothreitol (DTT), and eluted by a linear gradient of a second buffer containing 10 mM tris-HCl (pH 8.0), 1 M NaCl, and 1 mM DTT. Pooled fractions were concentrated to 10 to 20 mg/ml, and protein sample was flash-frozen by liquid nitrogen and stored at −80°C. For preparation of FAM46-BCCIP complexes, FAM46 and BCCIP proteins were incubated on ice with the ratio of 1:1 for 15 min before loading onto a Superdex 200 column equilibrated with gel filtration buffer [20 mM tris-HCl (pH 8.0), 150 mM NaCl, and 1 mM Tris (2-carboxyethyl) phosphine]. Pooled fractions were concentrated to 9 mg/ml, flash-frozen by liquid nitrogen, and stored at −80°C. GST-tagged proteins were expressed in the same strain, purified with a GST column containing Glutathione Sepharose 4B resin (17075601, Cytiva) and a Superdex 200 column, and stored at −80°C.

### PAP activity assay

Fluorescein-labeled 15-mer poly(A) oligos were synthesized (Integrated DNA Technologies) and dissolved in diethyl pyrocarbonate–treated water (R0603, Thermo Fisher Scientific). The reaction system of poly(A) polymerization assay contained the RNA oligo (5 mM), ATP (1 mM), MnCl_2_ (0.5 mM), ribonuclease inhibitor (AM2694, Thermo Fisher Scientific), FAM46D (5 mM), 25 mM tris-HCl (pH 7.0), 150 mM KCl, 0.2 mM DTT, and 10% glycerol. For assays including BCCIPα or BCCIPβ, the molar ratio between FAM46D and BCCIPα/BCCIPβ was 1:1. The reaction mixture was incubated at 30°C for 15 min, and the reaction was stopped by adding the TBE-Urea Sample Buffer (2×) (LC6876, Invitrogen). RNA products were loaded to 15% TBE-Urea Gel (EC68755BOX, Invitrogen). Gel images were taken with the Bio-Rad ChemiDoc MP Imaging System.

### Coimmunoprecipitation of FAM46A/FAM46C and BCCIPα/BCCIPβ

Full-length human FAM46A/FAM46C with a FLAG-tag and BCCIPα/BCCIPβ with a Myc-tag were cloned into the pRK5 vector (556104, BD PharMingen). The plasmids were transfected into HEK293T cells with Lipofectamine 3000 (L3000001, Invitrogen) according to the manufacturer’s instruction. Cells were harvested 36 hours after transfection and lysed in the lysis buffer containing 50 mM tris (pH 8.0), 150 mM NaCl, 0.1% NP-40, 1 mM DTT, and protease inhibitor (78442, Sigma-Aldrich). Lysates were cleared by centrifugation at 15,000 rpm for 10 min at 4°C. Anti-FLAG beads (A2220, Sigma-Aldrich) were added to the supernatant, and the mixture was rotated at 4°C for 1 hour. The beads were washed with a lysis buffer three times. Proteins remaining on the beads were resolved with 4 to 20% gradient SDS-PAGE (XP04205BOX, Invitrogen) and detected by Western blot using anti-FLAG (F7425, Sigma-Aldrich) and anti-Myc antibodies (2278, Cell Signaling Technology).

### In vitro pulldown binding assay

GST-tagged FAM46 proteins at 2 μM were incubated with 10 μl of Glutathione Sepharose 4B resin for 1 hour at 4°C and washed with TBST buffer [20 mM tris-HCl (pH 8.0), 150 mM NaCl, and 0.05% Tween 20] three times. BCCIP proteins at 2 μM were added, incubated for 2 hours at 4°C, and washed with TBST buffer three times. Samples were analyzed by SDS-PAGE gels.

### Nanobody screening

For nanobody screening, an Avi-tag (sequence: GLNDIFEAQKIEWHE) was added to the N terminus of FAM46C (residues 15 to 350). Biotin was covalently linked to the Avi-tag by the biotin ligase BirA as described previously (*48*). Screening for nanobodies that bind the FAM46C/BCCIPα-ΔS complex by magnetic-activated cell sorting (MACS) was performed as described previously with minor modification (*43*). Briefly, 2 × 10^10^ yeast cells expressing the nanobodies, induced in SD-Trp galactose medium [3.8 g of Trp drop-out medium supplement, 6.7 g of yeast nitrogen base, 10 ml of penicillin-streptomycin (stock at 10,000 U/ml), and 20 g of galactose], were washed and resuspended in selection buffer [20 mM Hepes (pH 7.5), 150 mM sodium chloride, 0.1% (w/v) bovine serum albumin, and 5 mM maltose]. Resuspended cells were incubated with Streptavidin MicroBeads (130-048-101, Miltenyi Biotec) at 4°C for 30 min. The mixture was flowed through the LS column (130-042-401, Miltenyi Biotec) as a preclear step to remove nanobodies that bind to the magnetic beads nonspecifically. Yeast cells in the flow through were washed and incubated with the biotin-labeled FAM46C/BCCIPα-ΔS complex (1 μM) at 4°C for 1 hour. Cells were then washed and incubated with Streptavidin MicroBeads at 4°C for 20 min. The mixture was loaded to the LS column in the magnetic assembly, and the column was washed extensively to remove unbound yeast cells and proteins. To elute bound yeast cells, the column was removed from the magnetic assembly and flushed with 7 ml of selection buffer. The eluted cells were collected and recovered for 24 hours in SD-Trp glucose medium (3.8 g of Trp drop-out medium supplement, 6.7 g of yeast nitrogen base, 100 U of penicillin-streptomycin, and 20 g of glucose in 1 liter). These cells (1 × 10^9^) were induced for protein expression again and subjected to the second- and third-round selection. The concentrations of FAM46C/BCCIPα-ΔS complex were decreased to 200 and 40 nM for the second and third round selection, respectively. After MACS selection, yeast cells were diluted and plated to generate single colonies. Plasmids from 20 single yeast colonies were extracted and sequenced. Unique nanobody sequences were cloned into a modified pET28 vector for expression in *E. coli*. A total of 20 nanobodies were expressed in the SHuffleT7 Express strain (C3029J, New England Biolabs) and purified with affinity chromatography followed by gel filtration chromatography. The interactions between nanobodies and the FAM46C/BCCIPα complex were validated by pulldown and gel filtration experiments. Nb.1 (sequence: QVQLQESGGGLVQAGGSLRLSCAASGTISPRGVMGWYRQAPGKEREFVAAINYGGTTYYADSVKGRFTISRDNAKNTVYLQMNSLKPEDTAVYYCAVYYYINSQRKVLLYWGQGTQVTVSS) was chosen for its good binding affinity to the FAM46C/BCCPα complex. The FAM46C/BCCIPα/Nb.1 complex was formed by incubating the three proteins with the ratio of 1:1:2 on ice for 15 min. The complex was further purified by a Superdex 200 column in a gel filtration buffer [20 mM tris (pH 8.0) and 150 mM NaCl], and peak fractions were pooled and concentrated to 5.7 mg/ml for cryo-EM grid preparation.

### Immunofluorescence of cellular localization of FAM46 and BCCIPα

FAM46 and BCCIP were cloned into modified pCMV5 vectors, encoding FLAG-tagged FAM46 and Myc-tagged BCCIP proteins, respectively. U2OS cells were seeded on glass coverslips in six-well plates (50,000 to 100,000 cells per well) with McCoy’s 5A (modified) medium (16600082, Thermo Fisher Scientific) supplemented with 10% fetal bovine serum (26140079, Thermo Fisher Scientific). After 24 hours, plasmids (200 to 300 ng per well) were transfected into cells with the Lipofectamine 3000 transfection reagent (L3000001, Thermo Fisher Scientific) to overexpress FLAG-tagged FAM46 or Myc-tagged BCCIPα proteins. Twenty-four hours after transfection, cells were washed with phosphate-buffered saline (PBS), fixed in 4% paraformaldehyde, and probed with indicated antibodies. Nuclei were stained with DAPI (4′,6-diamidino-2-phenylindole). Images were taken from random fields of coverslips by using a DeltaVision fluorescence microscope with a 40× objective, controlled with the software package SoftWoRx 7.0. The experiment for each sample was repeated three times. Images were processed with ImageJ and QuickFigures (*49*, *50*). Data were plotted with GraphPad Prism 9.

### Crystallization and structure determination

Initial crystallization screens were performed in 96-well plates by sitting-drop vapor diffusion, and crystallization optimizations were conducted by hanging-drop vapor diffusion. For the FAM46A/BCCIPα-ΔS complex, referring to FAM46A (residues 64 to 394)–BCCIPα (residues 50 to 322 with deletions of residues 112 to 122, 235 to 243, and 273 to 276), single crystals were obtained with sample (6 mg/ml) by seeding in the conditions of 0.1 M tris (pH 7.5), 0.4% (v/v) Jeffamine ED-2001, and 0.5 to 0.6 M sodium malonate. Crystals were cryo-protected in the buffer supplemented with 25% glycerol and flash-cooled in liquid nitrogen. For the FAM46A/BCCIPα-ΔL complex, referring to FAM46A (residues 64 to 394)–BCCIPα (residues 50 to 322 with deletions of residues 112 to 122 and 231 to 280), single crystals were obtained with sample (7.5 mg/ml) in the conditions of 0.1 M tris (pH 8.0), 0.2 M sodium thiocyanate, and 15 to 18% PEG 3350 (polyethylene glycol, molecular weight 3350). Crystals were cryo-protected in the buffer supplemented with 20% ethylene glycol and flash-cooled in liquid nitrogen.

Diffraction data were collected at Beamline 19ID at the advanced photon source (Argonne, IL). Data were processed with the HKL3000 software (*51*). Datasets with the resolutions of 3.5 and 3.2 Å were obtained for FAM46A/BCCIPα-ΔS and FAM46A/BCCIPα-ΔL, respectively. With the 3.5-Å dataset of the FAM46A/BCCIPα-ΔS complex, the structure was solved by molecular replacement using the FAM46C structure (PDB ID: 6W36) as the search model. The initial density map from this molecular replacement solution showed clear density for BCCIPα, which was built manually in Coot 0.98 (*52*). The structure of the FAM46A/BCCIPα-ΔL complex was determined by molecular replacement using the FAM46A/BCCIPα-ΔS structure as a searching model. Model building and refinement were conducted in Coot 0.98 and Phenix 1.19 (*53*), respectively. In the FAM46A/BCCIPα-ΔS structure, residues 64 to 169 and 179 to 392 of FAM46A and residues 57 to 93, 123 to 206, 214 to 225, and 293 to 322 of BCCIPα were successfully built. Residues 64 to 391 of FAM46A and residues 57 to 90, 121 to 206, 214 to 226, and 289 to 322 of BCCIPα were built in the FAM46A/BCCIPα-ΔL structure. Statistics for data collection and structure refinement are summarized in table S1. Structure validation was done using Molprobity as a part of the Phenix package (*54*).

### Cryo-EM data collection and image processing

The FAM46C/BCCIPα/Nb.1 complex at 5.7 mg/ml in the gel filtration buffer containing 3 mM Fluorinated Fos-Choline-8 (Anatrace) was applied to glow-discharged Quantifoil R1.2/1.3 300-mesh gold holey carbon grid (Quantifoil, Micro Tools GmbH), blotted at 4°C (100% humidity), and plunged into liquid ethane using Mark IV Vitrobot (FEI). Micrographs were collected on a Titan Krios microscope (FEI) equipped with a K3 Summit detector (Gatan) in the super-resolution correlated double-sampling (CDS) mode, with the slit of the GIF-Quantum energy filter set to 20 eV. The nominal magnification was ×81,000, corresponding to the actual pixel size of 1.08 Å. The dose of electrons was about 60 e^−^/Å^2^, fractionated over 36 frames and 7.2 s. Motion correction and dose weighting of the micrographs were carried out using MotionCor2 (v1.2) (*55*). GCTF 1.08 was used for contrast transfer function (CTF) correction (*56*). Particles were initially picked with the Laplacian-of-Gaussian method in RELION 3 (*57*). Good two-dimensional (2D) class averages from these particles were then chosen as the references for the second round of particle picking in RELION. Particles were extracted with the box size of 200 pixels and subjected to 2D classification. The results from the 2D classification showed that the globular particles were highly heterogeneous and contained no high-resolution features. A small portion of the sample formed relatively long filaments on the micrographs, reminiscent of strings of Chinese lanterns. 2D class averages of particles segmented from the filaments were of much better quality. These particles were therefore selected for 3D classification, with the initial model generated using EMAN2 (*58*). Particles from the good 3D class were selected for 3D refinement. The initial map clearly showed the D2 symmetry, which was then applied during the subsequent refinement, leading to a final reconstruction of resolution 6.5 Å according to the “gold-standard” Fourier shell correlation (FSC) criterion (cutoff of 0.143).

The medium resolution of the cryo-EM map did not allow de novo atomic model building but clearly resolved α helices and β sheets in the proteins. Four copies of the crystal structure of BCCIPα solved in this study could be unambiguously docked into the cryo-EM density map. The model of Nb.1 was generated by using AlphaFold 2 as implemented in ColabFold (*36*, *37*) and docked into the map. The remaining density could not accommodate the full-length structure of human FAM46C (PDB ID: 6W36). More analyses suggested that the density contained four protomers of the N-terminal lobe of FAM46C, whereas the C-lobe was missing. There are two helices near the junction between two BCCIPα molecules. They do not belong to BCCIPα and may be a part of the C-terminal lobe of FAM46C, which may have been dislodged and pushed always by the binding of BCCIPα and Nb.1. There is additional density below these two helices, which constitute the connection between the individual lantern-shaped circular body of the structure. Because of the poor quality of the density, no atomic model was assigned to it. The model containing four copies of BCCIPα, the FAM46C N-terminal lobe, and Nb.1 was manually adjusted to resolve clashes in Coot 0.94 and rigid-body refined in Phenix 1.18 (*52*, *59*).

The data collection and structural refinement statistics are summarized in table S2. Molecular graphs were rendered in PyMOL (The PyMOL Molecular Graphics System, Schrödinger) and ChimeraX (*60*). Topology diagrams were generated with Pro-origami (*61*).

## Notes

View/request a protocol for this paper from *Bio-protocol*.
