# Supplementary material for: Inhibition of FAM46/TENT5 activity by BCCIPα adopting a unique fold
Source: Sci Adv. 2023 Apr 5;9(14):eadf5583. doi: 10.1126/sciadv.adf5583 (PMC10075960; doi:10.1126/sciadv.adf5583)
Supplement: Supplementary file 1 — Figs. S1 to S7 Tables S1 and S2 [file sciadv.adf5583_sm.pdf]

Science Advances
AAAS

Supplementary Materials for

Inhibition of FAM46/TENT5 activity by BCCIPα adopting a unique fold

Shun Liu *et al.*

Corresponding author: Xiao-Chen Bai, xiaochen.bai@utsouthwestern.edu;
Xuewu Zhang, xuewu.zhang@utsouthwestern.edu

Sci. Adv. **9**, eadf5583 (2023)
DOI: 10.1126/sciadv.adf5583

This PDF file includes:

Figs. S1 to S7
Tables S1 and S2

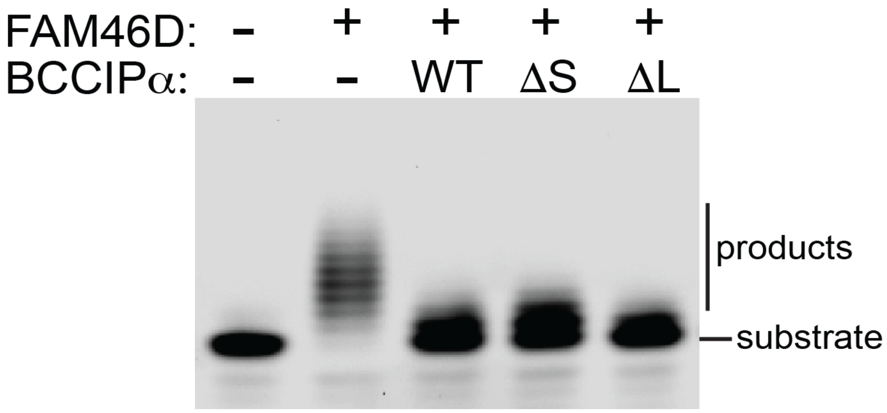

Figure S1. BCCIPα-WT, ΔS and ΔL show similar levels of inhibition on the PAP activity of FAM46D. The results shown are representative of three biological replicates.

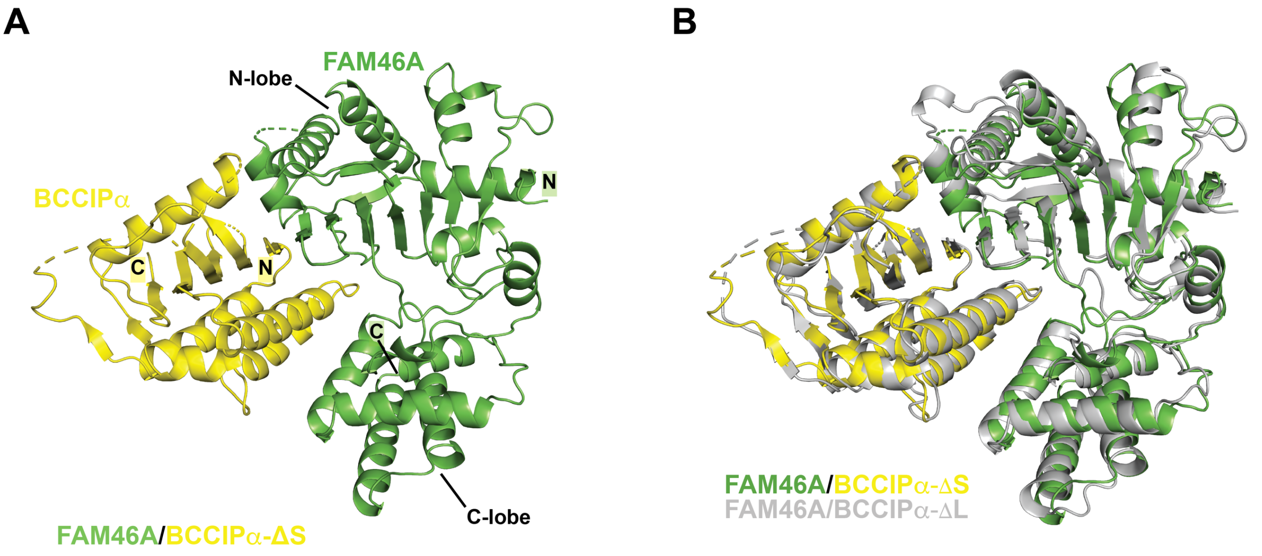

Figure S2. Crystal structure of the FAM46A/BCCIPα-ΔS complex. (**A**) Overall structure of the FAM46A/BCCIPα-ΔS complex. (**B**) Superimposition of the structures of the FAM46A/BCCIPα-ΔS and FAM46A/BCCIPα-ΔL complexes.

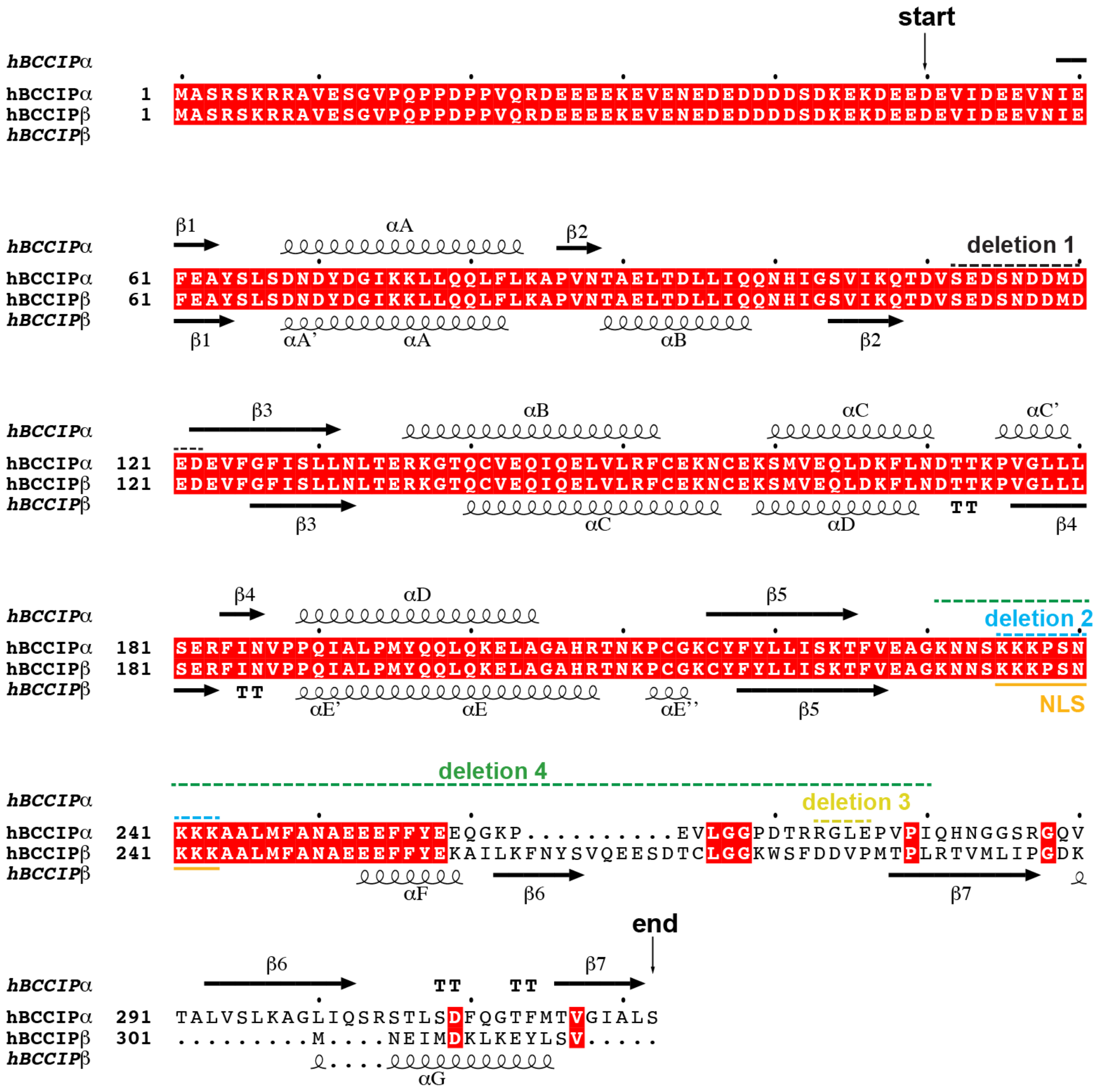

Figure S3. Sequence alignment of human BCCIPα and BCCIPβ. Secondary structure elements of BCCIPα and BCCIPβ are marked above and below the alignment, respectively. “Start” and “end” denote the boundaries of the BCCIPα constructs for protein expression. BCCIPα-ΔS contains deletions 1, 2 and 3. BCCIPα-ΔL contains deletions 1 and 4. NLS, nuclear localization signal.

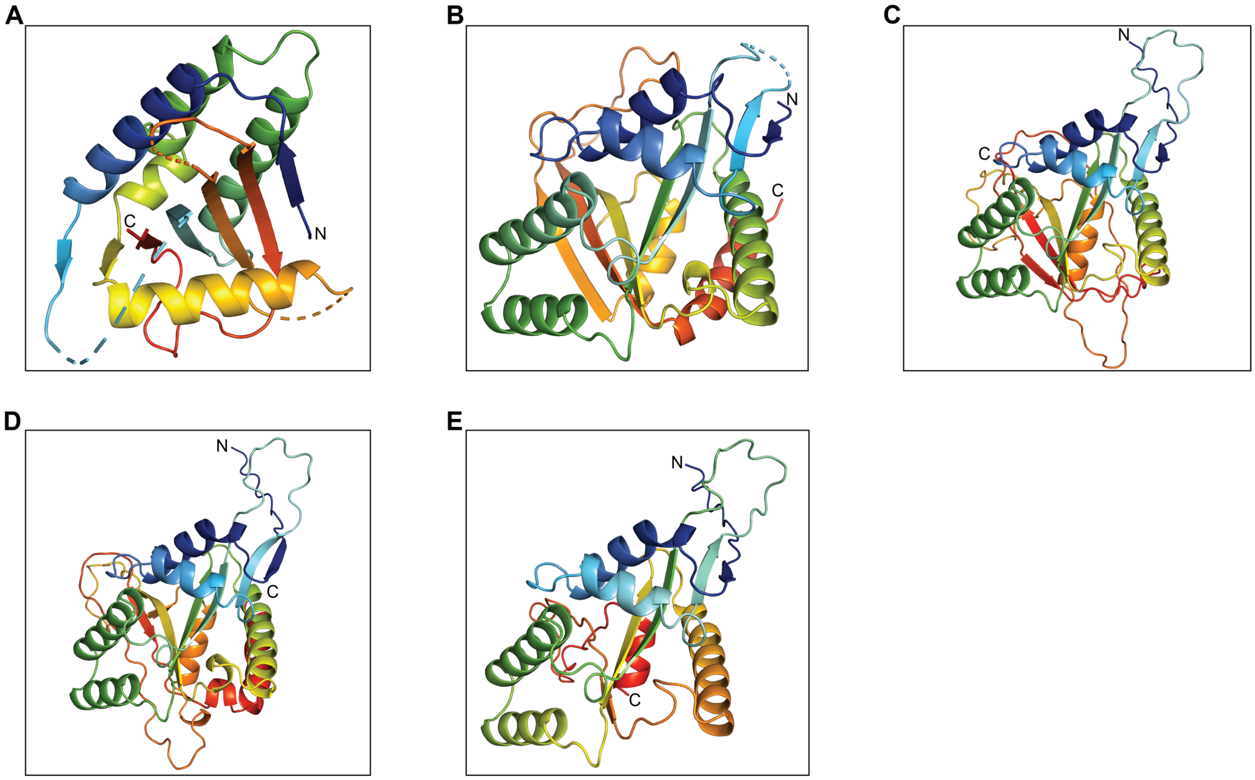

Figure S4. Comparison of crystal structures of BCCIPα and BCCIPβ, and predicted structures of BCCIPα. (**A**) Crystal structure of BCCIPα. (**B**) Crystal structure of BCCIPβ. (**C**) Predicted structure of BCCIPα by AlphaFold. (**D**) Predicted structure of BCCIPα by EMSFold. (**E**) Predicted structure of residues 1-258 common in both BCCIPα and BCCIPβ by AlphaFold. The N-terminal disordered region (residues 1-49) of BCCIPα is omitted in the figures. The structures are colored with the rainbow color scheme from the N- to C-termini. It is evident that all the predicted structures of BCCIPα resemble the crystal structure of BCCIPβ, rather than that of BCCIPα.

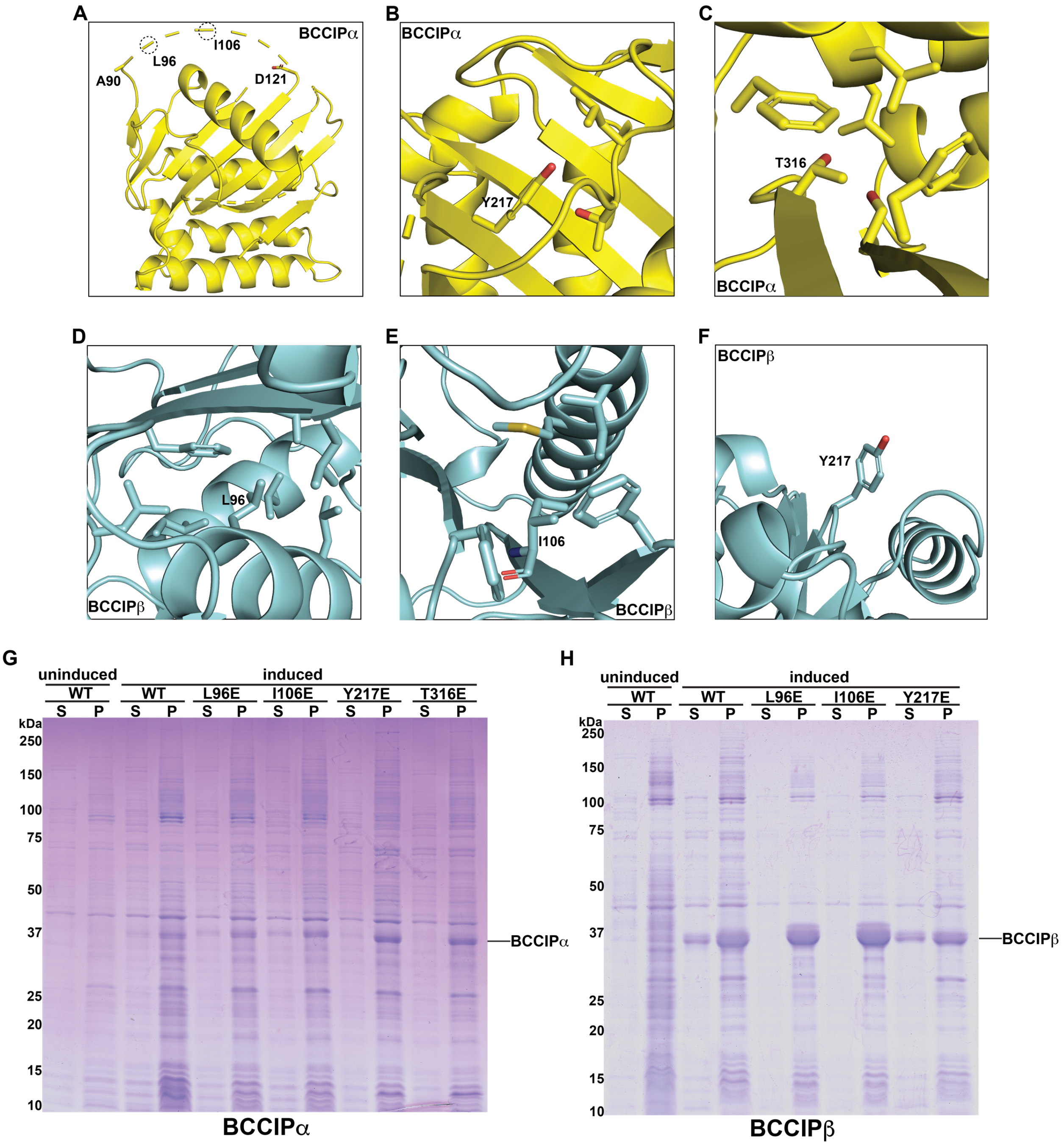

Figure S5. Mutational analyses of the distinct folds of BCCIPα and BCCIPβ. (**A-C**) and (**D-F**) Locations of residues chosen for mutational analyses in the structures of BCCIPα and BCCIPβ, respectively. (**G**) and (**H**) Effects of the mutations on the expression level and solubility of BCCIPα and BCCIPβ, respectively. The proteins were expressed in BL21(DE3) using the same protocol as for expressing of the wild type. Soluble (s) and pellet (p) fractions from the cells were analyzed with SDS-PAGE. For BCCIPα, L96E and I106E did not affect the expression or solubility, whereas Y217E and T316E increased the insoluble fraction but nearly eliminated the soluble protein. In contrast, for BCCIPβ, L96E and I106E abolished soluble protein expression, while Y217E behaved similarly to the wild type. These results are consistent with the roles of these residues in the structures of BCCIPα and BCCIPβ, respectively, as shown in (**A-F**).

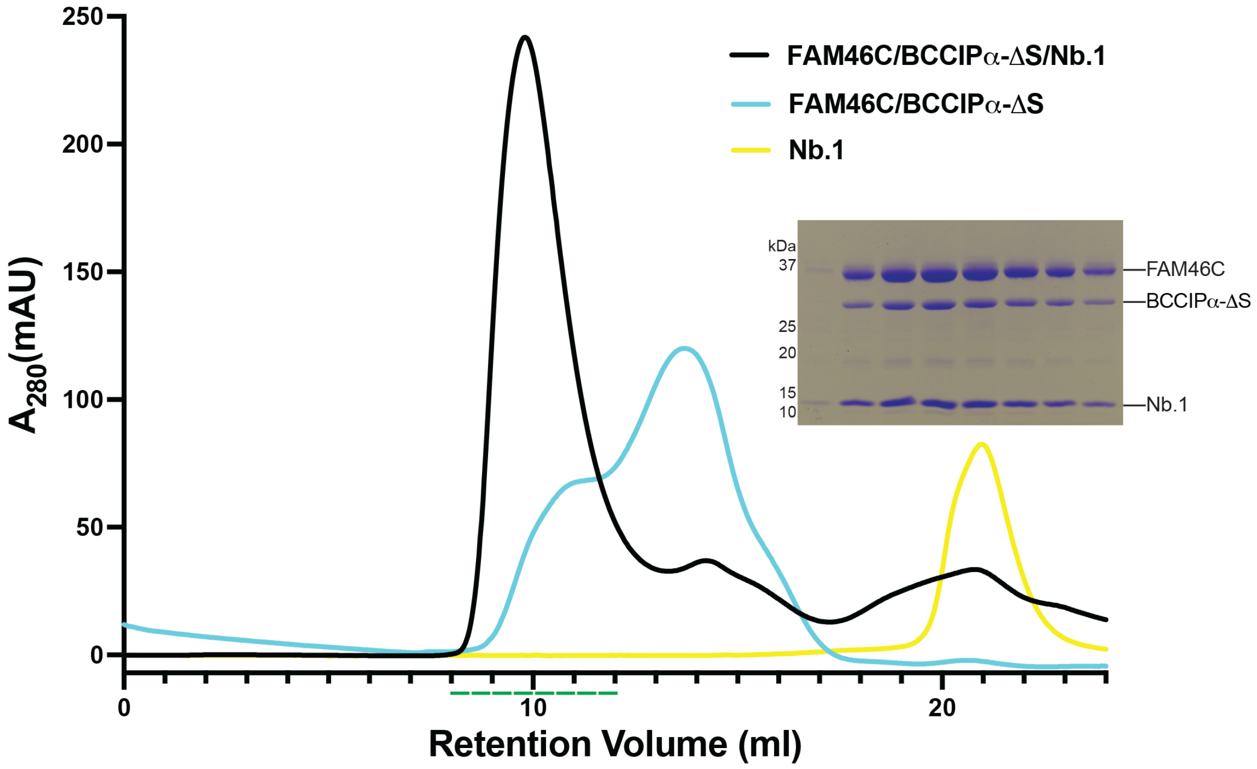

Figure S6. Gel filtration chromatography and SDS-PAGE analysis of the FAM46C/BCCIPα/Nb.1 complex. The complex peak is at ~10 ml, suggesting that it has a large molecular weight, which is consistent with the formation of oligomers of the complex seen in cryo-EM. Fractions indicated by the green bars at the bottom of the chromatogram were analyze by SDS-PAGE, showing co-elution of FAM46C, BCCIPα and Nb.1.

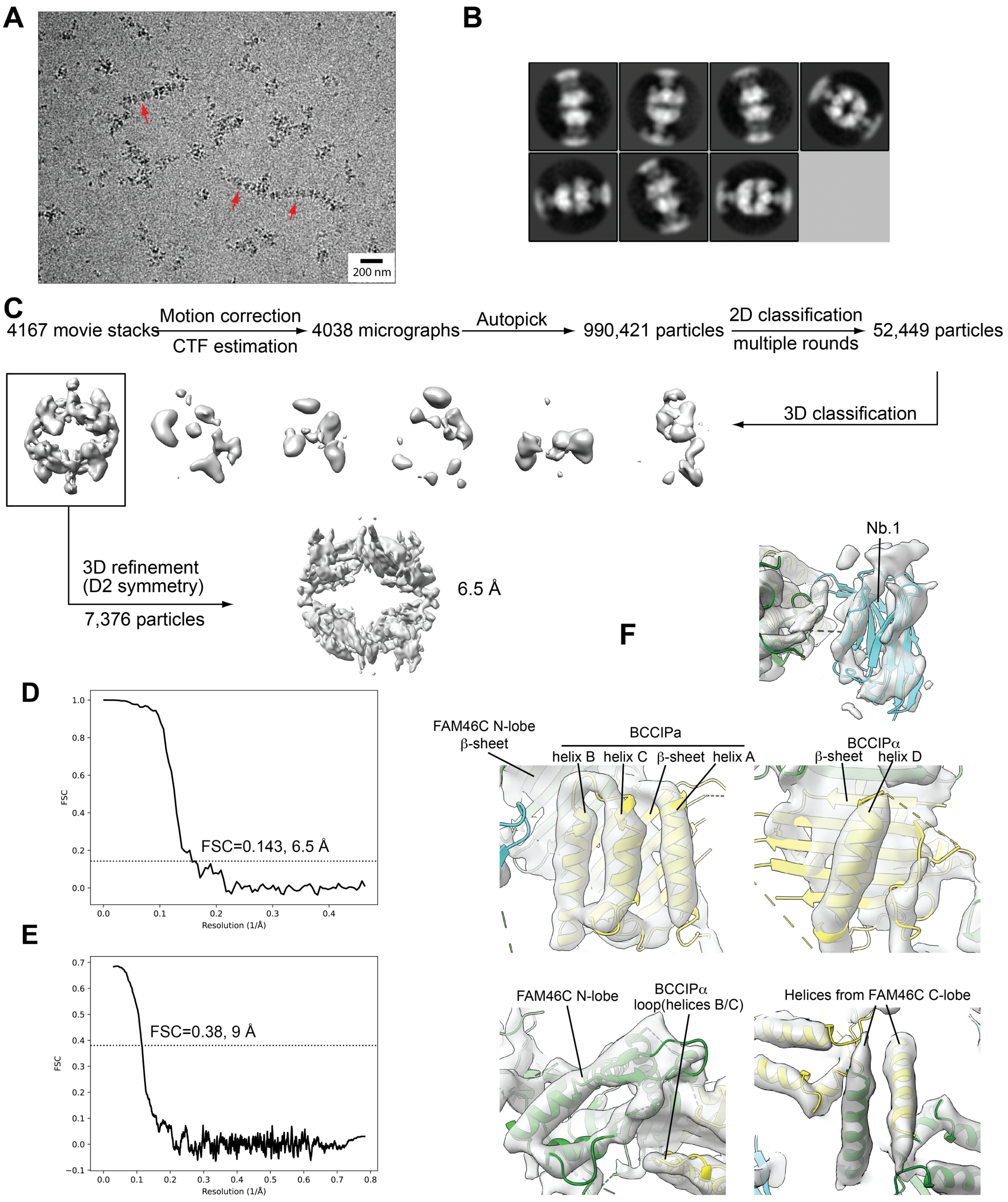

Figure S7. Cryo-EM image processing procedure of the FAM46C/BCCIPα/Nb.1 complex. (**A**) Motion-corrected micrograph. Arrows highlight filamentous oligomers of the complex. (**B**) 2D class averages. (**C**) Data processing procedure. (**D**) Fourier Shell Correlation (FSC) between the two half maps. (**E**) FSC between the map and the model. (**F**) Cryo-EM density of various parts of the structure.

Table S1. Data collection and crystal structure refinement statistics.

	FAM46A/BCCIPα-ΔS (PDB ID: 8EXE)	FAM46A/BCCIPα-ΔL (PDB ID: 8EXF)
**Data collection**		
Wavelength (Å)	0.979	0.979
Resolution (Å^2)	50.00-3.50 (3.56-3.50)	50.00-3.20 (3.56-3.20)
Space group	$P\ 2_12_12_1$	$C\ 222_1$
Unit cell dimensions		
a, *b*, *c* (Å)	87.80, 88.15, 102.98	91.62, 188.15, 93.34
α, *β*, *γ* (°)	90.0, 90.0, 90.0	90.0, 90.0, 90.0
Redundancy	4.1 (3.8)	3.9 (3.7)
Completeness (%)	96.3 (90.2)	98.9 (98.2)
Reflections (unique)	10, 371	13, 367
I/σ_I	7.9 (1.1)	17.8 (1.1)
R_{sym} (%)	16.5 (127.7)	7.0 (95.4)
R_{pim} (%)	8.8 (67.0)	4.0 (57.9)
$CC_{1/2}$	0.981 (0.521)	0.989 (0.462)
**Refinement**		
No. of non-hydrogen atoms	3, 877	3, 963
Protein	3, 877	3, 962
Water	-	1
Average *B* factor (Å^2)	37.8	52.9
Protein	37.8	52.9
Water	-	12.3
R_{work}/R_{free} (%)	26.04/29.74	20.71/24.94
RMSDs		
Bond length (Å)	0.004	0.004
Bond angle (°)	0.681	0.629
Favored/allowed/outliers (%)	91.74/8.26/0.00	95.06/4.94/0.00

Values for the highest resolution shell are given in parentheses.

Table S2. Cryo-EM structure data collection and structure refinement statistics.

Magnification	81,000
Voltage (kV)	300
Electron exposure ($e^-/Å^2$)	50
Defocus range (µm)	1.5-2.5
Pixel size (Å)	1.08
Symmetry imposed	D2
Initial particle images (no.)	990,421
Final particle images (no.)	7,376
Map resolution (Å)	6.5
FSC threshold	0.143
Initial model used (PDB code)	6w36
Model resolution (Å)	9.0
FSC threshold	0.38
Map sharpening B factor ($Å^2$)	-100
Model Composition	
Non-hydrogen atoms	14,740
Protein residues	1916
B factors ($Å^2$)	341.3
R.m.s. deviations	
Bond length (Å)	0.01
Bond angle (°)	1.266
Validation	
Molprobity score	1.85
Clashscore	9.3
Poor rotamers (%)	0.75
Ramachandran plot	
Favored (%)	94.8
Allowed (%)	5
Outliers (%)	0.2
